# The Aberrant Passage: A Rare Spontaneous Pancreatic Pseudocyst-Portal Vein Fistula

**DOI:** 10.7759/cureus.110883

**Published:** 2026-06-15

**Authors:** Menooa Simonian, Sushant Babbar, Akhil Baby, Rajesh Sasidharan

**Affiliations:** 1 Chicago Medical School, Rosalind Franklin University of Medicine and Science, North Chicago, USA; 2 Department of Radiodiagnosis, Dayanand Medical College and Hospital, Ludhiana, IND; 3 Center of Excellence in Gastrointestinal Sciences, Rajagiri Hospital, Kochi, IND

**Keywords:** endoscopic pancreatic duct stenting, hepatic lesion, pancreatic pseudocyst fistula, portal cavernoma, portal vein fistula, vascular complication of pancreatitis

## Abstract

A 45-year-old man with a history of alcohol-induced chronic pancreatitis presented with acute abdominal pain and low-grade fever. Initial imaging with contrast-enhanced CT showed a widened portal vein containing fluid that appeared different from a typical solid clot. Further specialised magnetic resonance imaging (MRCP) confirmed a fistula connecting the pancreatic duct directly to the portal vein. On that MRCP imaging, there's evidence of intravascular fluid collection within the portal vein showing homogeneous T2-hyperintense signal characteristics with the adjacent pancreatic fluid, suggesting direct extension. An axial T2-weighted MRI confirmed the existence of the fistula. A review of a previous MRCP performed six months prior showed a pancreatic head pseudocyst already extending into the portal vein, suggesting that the fistula developed gradually over several months. The diagnosis was confirmed by sampling the fluid within the vein, which showed significant levels of pancreatic enzymes.

## Introduction

Chronic pancreatitis is characterized by progressive inflammatory damage; however, the formation of pseudocysts - which are encapsulated collections of amylase-rich fluid lacking a true epithelial lining - most frequently arises as a sequela of acute or acute-on-chronic pancreatitis [1]. As active pancreatic secretions continue to accumulate within these non-epithelial walls, hydrostatic pressure increases, causing these encapsulated collections to infiltrate through fascial planes and eventually erode neighboring vascular structures [2]. Vascular complications of chronic pancreatitis frequently include venous thrombosis or arterial pseudoaneurysms [3,4]; however, a pancreatic pseudocyst-portal vein fistula is a rare pathology signified by a direct communication between the pancreatic ductal system and the venous circulation. Diagnosing this condition on a routine CT scan is often challenging due to potential misidentification of dense, proteinaceous fluid or necrotic debris within the vessel as simple portal vein thrombosis (PVT), as both pathologies present as low-attenuation filling defects that obscure the normal contrast enhancement of the vein. As such, it is critical to employ further imaging, such as MRI, to avoid missing the diagnosis, which carries the potential for systemic dissemination of pancreatic enzymes that may result in widespread systemic fat necrosis or multi-organ failure.

## Case presentation

A 45-year-old male with a history of alcohol-induced chronic pancreatitis presented with acute abdominal pain and a low-grade fever. On admission, the patient was hemodynamically stable with a temperature of 38.1°C, a pulse rate of 96 beats per minute, and a blood pressure of 118/76 mmHg. Abdominal examination revealed epigastric tenderness without guarding, rebound, or clinical jaundice.

Initial laboratory evaluation showed a hemoglobin of 11.2 g/dL, a total leukocyte count of 14.8 × 10⁹/L with 82% neutrophils, and a platelet count of 108 × 10⁹/L; the thrombocytopenia was consistent with hypersplenism related to an underlying portal cavernoma and portal hypertension. Inflammatory markers were elevated, featuring a C-reactive protein (CRP) of 96 mg/L and an erythrocyte sedimentation rate (ESR) of 48 mm in the first hour, whereas serum procalcitonin remained low at 0.3 ng/mL, arguing against overt bacterial sepsis.

Pancreas-specific enzymes were raised, with a serum amylase of 142 U/L (reference <100 U/L) and a serum lipase of 680 U/L (reference <60 U/L), in keeping with active pancreatic inflammation. Liver function tests demonstrated a mildly elevated total bilirubin of 1.6 mg/dL, an alkaline phosphatase (ALP) of 168 U/L, and a gamma-glutamyl transferase (GGT) of 214 U/L, with aspartate aminotransferase (AST) and alanine aminotransferase (ALT) values of 64 U/L and 48 U/L, respectively. This pattern was attributable to chronic alcohol use rather than biliary obstruction, which agreed with the separately visualized, normal common bile duct on magnetic resonance cholangiopancreatography (MRCP). Serum albumin was reduced to 3.1 g/dL, while renal function, serum calcium, and the coagulation profile were within normal limits.

Diagnostic evaluation involved multi-modal imaging to delineate the vascular-ductal anatomy. Following the imaging findings, aspiration of the portal venous cystic content was performed. This yielded brownish-grey fluid with a markedly elevated amylase of 2,400 U/L and lipase of 8,600 U/L. These fluid values far exceeded their serum counterparts, confirming a direct communication between the pancreatic ductal system and the portal venous lumen.

**Figure 1:**
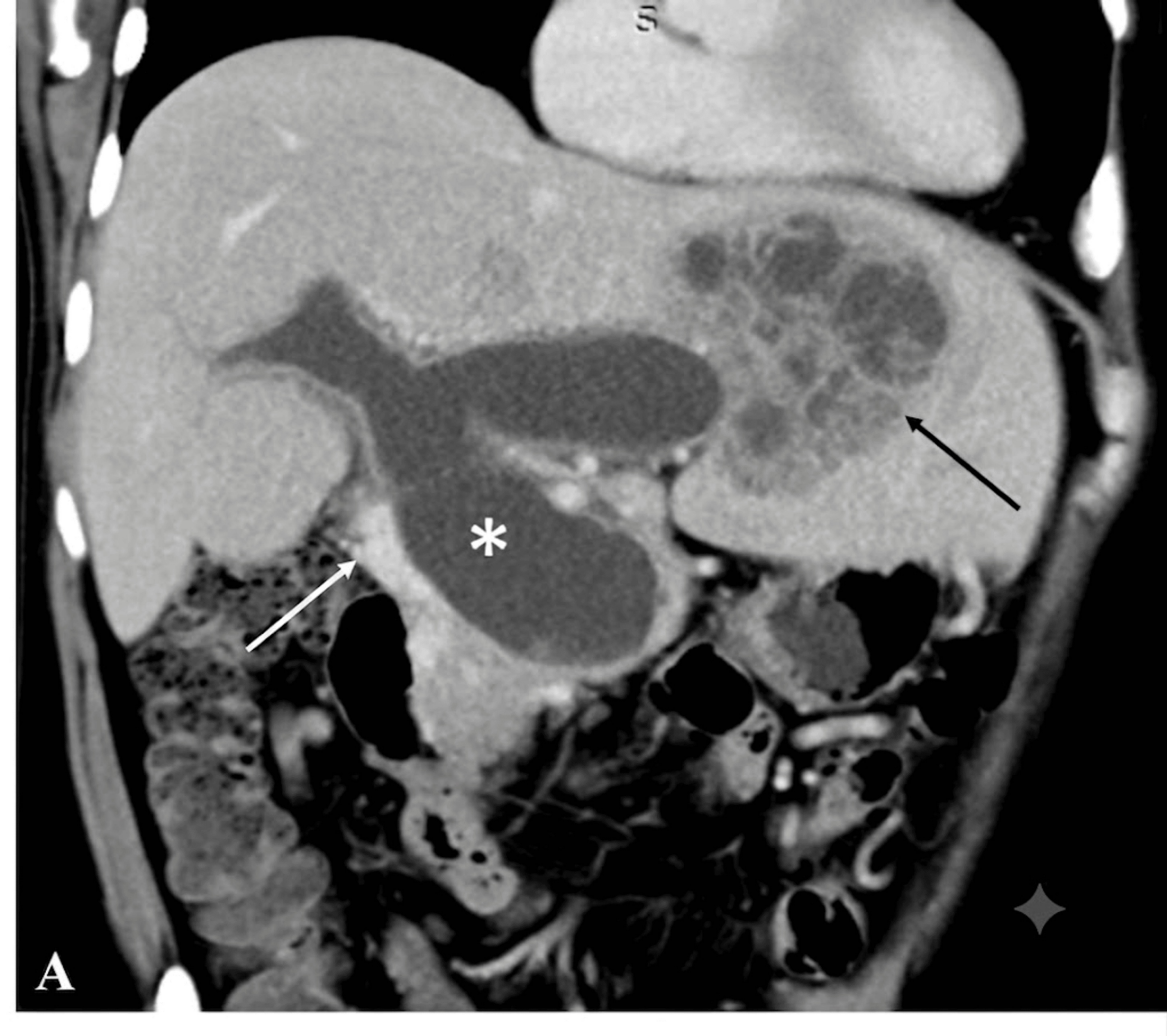
Contrast-enhanced CT demonstrating a pancreatico-portal fistula with portal vein distension and cavernous transformation. The white asterisk indicates cystic contents within the distended portal vein; the thin black arrow indicates a multiseptated cystic lesion in the left hepatic lobe; the thin white arrow indicates a portal cavernoma.

Contrast-enhanced CT (Figure 1) revealed a distended portal vein (PV) containing cystic contents without intraluminal contrast opacification, along with a multiseptated cystic lesion in the left hepatic lobe, which is in contrast to the expected hyperdensity characteristic of a solid thrombus. Additionally, portal cavernoma and prominent perigastric collaterals were noted on this CT image; those two findings in particular signify that the portal venous flow may have become chronically compromised, resulting in the formation of several periportal venous bypasses.

**Figure 2:**
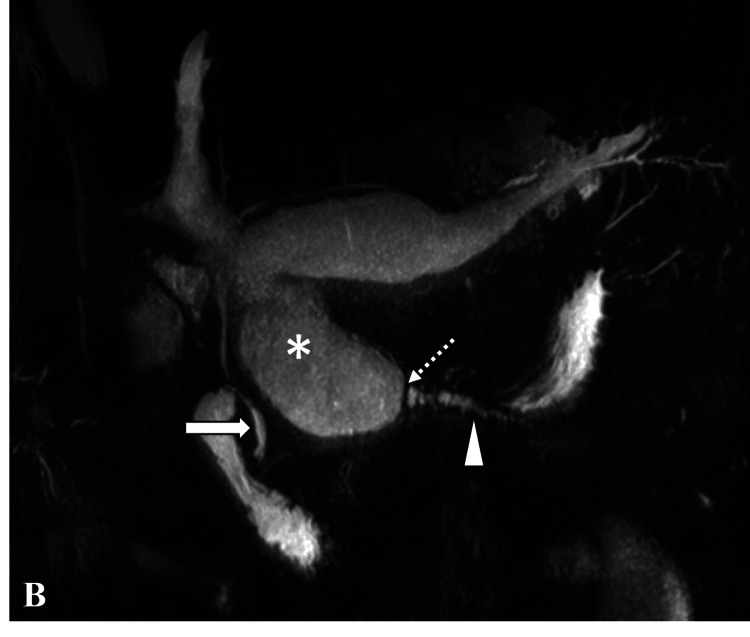
MRCP demonstrating a fistulous communication between the main pancreatic duct and the portal vein. Dashed white arrow indicates the fistulous communication; white arrowhead indicates the dilated main pancreatic duct; thick white arrow indicates the common bile duct; white asterisk indicates cystic contents within the distended portal vein.

**Figure 3:**
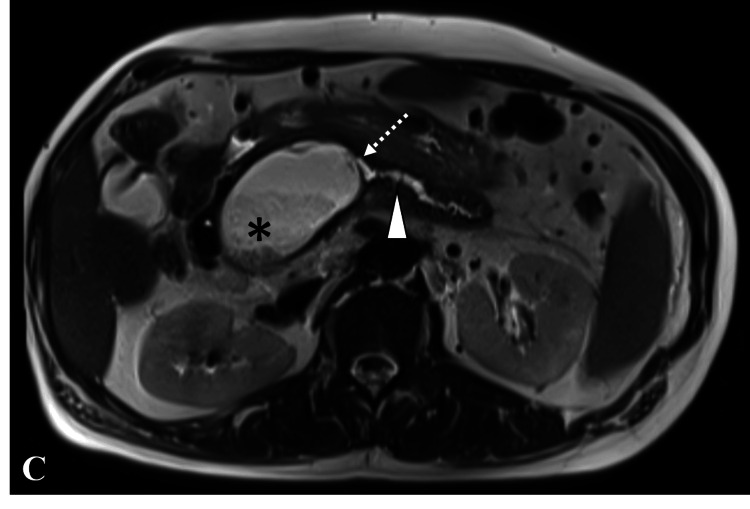
Axial T2-weighted MRI confirming a pancreatico-portal fistula with intraluminal portal vein debris. Dashed white arrow indicates the pancreatico-portal fistulous communication; black asterisk indicates dependent debris within the portal vein; white arrowhead indicates the dilated main pancreatic duct.

An MRCP (Figure 2) demonstrated a fistulous communication between the dilated main pancreatic duct and the PV. Critically, the signal intensity of the intravascular fluid was similar to that of the fluid within the pancreatic duct, likely pointing to a similar material between the two structures. The common bile duct (CBD) was visualized separately, which confirmed that the fistula was contained between the pancreatic duct and the portal vein interface. An axial T2-MRI (Figure 3) confirmed this communication along with dependent debris within the vein. 

**Figure 4:**
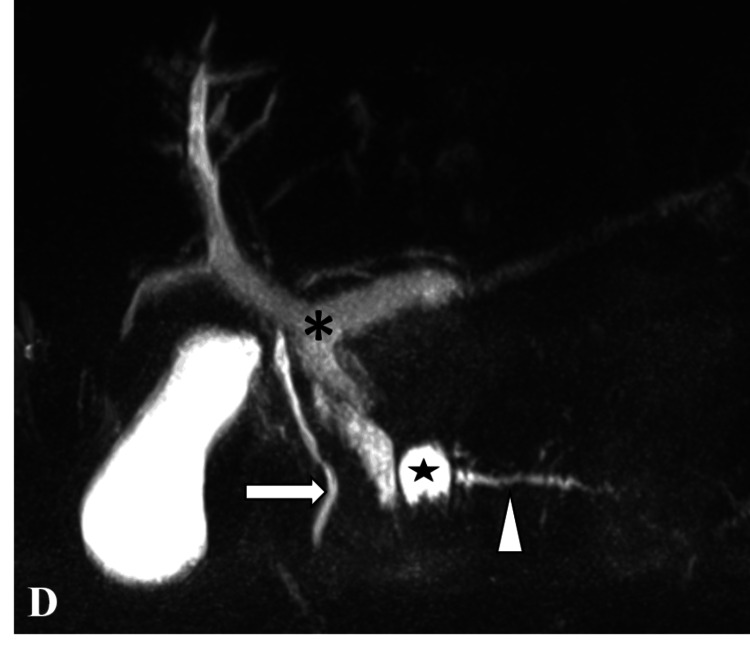
Historical MRCP (six months prior) showing an eroding pancreatic head pseudocyst tracking toward the portal vein. Black star symbol indicates the pancreatic head pseudocyst; black asterisk indicates dependent debris within the portal vein; thick white arrow indicates the common bile duct; white arrowhead indicates the dilated main pancreatic duct.

MRCP performed six months prior (Figure 4) showed a pancreatic head pseudocyst already extending into the portal vein, indicating that the fistula had likely resulted from a subacute, enzymatic erosion as opposed to a sudden rupture of the pseudocyst contents. Diagnosis was biochemically confirmed through percutaneous aspiration of the portal vein, which revealed markedly elevated amylase and lipase levels. Although scheduled for endoscopic ductal stenting to bypass the fistula, the patient was lost to follow-up.

## Discussion

The formation of a pancreatic pseudocyst-portal vein fistula requires two concurrent pathological states: ductal hypertension and enzymatic erosion. The pseudocyst itself may produce a high-pressure gradient that drives enzyme-rich fluid against the vessel wall [5]. Pancreatic enzymes then may deteriorate the structural proteins of the venous media and adventitia, ultimately causing a transmural breach [6]. A rather unique aspect of this case is the finding of the portal cavernoma, which suggests that the fistula-induced inflammatory reaction likely catalyzed a localized phlebitis and thrombosis that eventually recanalized through collateralization over time. This cavernoma may have even acted as a hemodynamic buffer, possibly restricting the volume of pancreatic enzymes from penetrating the systemic circulation.

Arguably, the greatest pitfall in managing such patients is potentially dismissing the cystic portal vein contents found on a CT scan as a simple necrotic thrombus. Therefore, MRCP is a crucial diagnostic modality to better correlate the signal of the intravascular fluid with the pancreatic ductal contents. Minimally invasive endoscopic drainage is the preferred treatment for symptomatic or complicated pseudocysts because it achieves a remarkably high technical success rate and overall long-term resolution rate without the need for external drains. Depending on ductal communication and proximity to the gastrointestinal tract, decompression is achieved via a transpapillary approach using ERCP or a transmural (transgastric or transduodenal) approach guided by endoscopic ultrasound to safely avoid intervening vessels. Furthermore, clinical outcomes are optimized, and complications like hemorrhage are minimized by utilizing double-pigtail stents rather than straight stents to prevent cyst wall erosion [4].

## Conclusions

Spontaneous pancreatic pseudocyst-portal vein fistulas require timely diagnosis and targeted intervention. This case provides several important clinical takeaways. The first is that a pancreatic pseudocyst that is in contact with a major vessel requires proactive monitoring to avoid fistula formation. Equally important is that the incidental discovery of cavernous transformation in a pancreatitis patient should inform the urgency of finding a possible vascular fistula. Early recognition and management of this pathology are critical to prevent systemic enzyme-mediated organ failure.
